# Systematic analysis of the BET family in adrenocortical carcinoma: The expression, prognosis, gene regulation network, and regulation targets

**DOI:** 10.3389/fendo.2023.1089531

**Published:** 2023-01-30

**Authors:** Yongli Situ, Quanyan Liang, Ziying Zeng, Jv Chen, Zheng Shao, Qinying Xu, Xiaoyong Lu, Yongshi Cui, Juying Zhang, Lingling Lu, Li Deng

**Affiliations:** ^1^ Department of Parasitology, Guangdong Medical University, Zhanjiang, Guangdong, China; ^2^ Department of Pharmacy, Affiliated Hospital of Guangdong Medical University, Zhanjiang, China

**Keywords:** BRD4, adrenocortical carcinoma, target prediction, gene regulation network, PFI-1, *BRD3*, *BRD2*

## Abstract

**Background:**

Bromodomain and extracellular terminal (BET) family (including BRD2, BRD3, and BRD4) is considered to be a major driver of cancer cell growth and a new target for cancer therapy. Currently, more than 30 targeted inhibitors have shown significant inhibitory effects against various tumors in preclinical and clinical trials. However, the expression levels, gene regulatory networks, prognostic value, and target prediction of *BRD2*, *BRD3*, and *BRD4* in adrenocortical carcinoma (ACC) have not been fully elucidated. Therefore, this study aimed to systematically analyze the expression, gene regulatory network, prognostic value, and target prediction of *BRD2*, *BRD3*, and *BRD4* in patients with ACC, and elucidated the association between BET family expression and ACC. We also provided useful information on *BRD2*, *BRD3*, and *BRD4* and potential new targets for the clinical treatment of ACC.

**Methods:**

We systematically analyzed the expression, prognosis, gene regulatory network, and regulatory targets of *BRD2*, *BRD3*, and *BRD4* in ACC using multiple online databases, including cBioPortal, TRRUST, GeneMANIA, GEPIA, Metascape, UALCAN, LinkedOmics, and TIMER.

**Results:**

The expression levels of *BRD3* and *BRD4* were significantly upregulated in ACC patients at different cancer stages. Moreover, the expression of *BRD4* was significantly correlated with the pathological stage of ACC. ACC patients with low *BRD2*, *BRD3*, and *BRD4* expressions had longer survival than patients with high *BRD2*, *BRD3*, and *BRD4* expressions. The expression of *BRD2*, *BRD3*, and *BRD4* was altered by 5%, 5%, and 12% in 75 ACC patients, respectively. The frequency of gene alterations in the 50 most frequently altered *BRD2*, *BRD3*, and *BRD4* neighboring genes in these ACC patients were ≥25.00%, ≥25.00%, and ≥44.44%, respectively. *BRD2*, *BRD3*, and *BRD4* and their neighboring genes form a complex network of interactions mainly through co-expression, physical interactions, and shared protein domains. Molecular functions related to *BRD2*, *BRD3*, and *BRD4* and their neighboring genes mainly include protein-macromolecule adaptor activity, cell adhesion molecule binding, and aromatase activity. Chemokine signaling pathway, thiamine metabolism, and olfactory transduction were found to be enriched as per the KEGG pathway analysis. SP1, NPM1, STAT3, and TP53 are key transcription factors for *BRD2*, *BRD4*, and their neighboring genes. MiR-142-3P, miR-484, and miR-519C were the main miRNA targets of *BRD2*, *BRD3*, BRD4, and their neighboring genes. We analyzed the mRNA sequencing data from 79 patients with ACC and found that *ZSCAN12*, *DHX16*, *PRPF4B*, *EHMT1*, *CDK5RAP2*, *POMT1*, *WIZ*, *ZNF543*, and *AKAP8* were the top nine genes whose expression were positively associated with *BRD2*, *BRD3*, and *BRD4* expression. The expression level of *BRD2*, *BRD3*, and *BRD4* positively correlated with B cell and dendritic cell infiltration levels. *BRD4*-targeted drug PFI-1 and (*BRD2*, *BRD3*, and *BRD4*)-targeted drug I-BET-151 may have good inhibitory effects on the SW13 cell line.

**Conclusions:**

The findings of this study provide a partial basis for the role of *BRD2*, *BRD3*, and *BRD4* in the occurrence and development of ACC. In addition, this study also provides new potential therapeutic targets for ACC, which can serve as a reference for future basic and clinical research.

## 1 Introduction

Adrenal cortical carcinoma (ACC) is a sporadic adrenocortical endocrine tumor with an annual incidence of 0.5 to 2 cases per million. The number of cases in the female population generally outnumbers that of the male population (1.5:1) (1, 2). The clinical manifestations of ACC can be in three different forms. Symptoms related to hormone excess occur in 60% of the patients with ACC. Another 20% of patients with ACC have abdominal pain or fullness due to tumor growth, and the remaining 20% ​​ have unrelated symptoms detected by abdominal imaging (3–5). The prognosis of ACC is also extremely poor since the clinical presentation of ACC is often difficult to determine, and most patients are diagnosed in the late metastatic stage of the disease. Although ACC patients with the locoregional disease are treated with surgery, approximately 75% of patients experience recurrence after treatment (6). In addition, for advanced metastatic disease, the median overall survival was 12–15 months, and the 5-year overall survival was <15% (7, 8). Patients with ACC did not benefit from improvements in overall tumor treatment when compared to those with other tumor types. Evidence suggests that chemotherapy and radiation therapy are ineffective in most ACC cases. Mitotane, the only drug currently approved by the U.S. Food and Drug Administration for ACC, is usually only temporarily effective, and its use is often accompanied by significant side effects (9, 10). Complete tumor resection remains the only curative treatment (11). Therefore, in the current situation of uncertain therapeutic effects, limited toxic drugs, and high risk of disease recurrence, it is important to systematically analyze targeted prediction and prognostic markers in patients with ACC.

Bromodomain and extracellular terminal (BET) family consists of four members, including BRD2, BRD3, BRD4, and BRDT, which play key roles in various cell processes, including cell cycle, apoptosis, migration, and invasion (12). Therefore, the BET family can enhance its carcinogenic function by increasing its expression or promoting the transcriptional activity of carcinogenic factors. BET family is over-expressed in a variety of human cancers, which are closely related to human carcinogenesis (13). They are considered to be attractive therapeutic targets for selectively inhibiting cancer patients (14). However, BRDT is primarily expressed in germ cells, and BRD2, BRD3, and BRD4 are ubiquitously expressed. Among them, BRD4 is the most extensively studied in tumor research. BRD2, BRD3, and BRD4 can specifically recognize acetylated lysine residues and regulate the replication and transcription of related genes, thereby affecting key processes in cell carcinogenesis (15). They have two tandem bromodomains (BD1 and BD2) that regulate gene expression by binding to promoters and super-enhancer regions and promoting transcriptional elongation in cancer. The inhibition of BRD2, BRD3, and BRD4 shortens signaling between super-enhancer regions and oncogene target promoters, resulting in cell-specific inhibition of oncogene expression and ultimately cancer cell death. They contain multiple acetylated residues and activate transcription by binding to acetylated lysine residues on target proteins and mediating chromatin decompression (16). Studies have reported that *BRD4* inhibitors treat cancer by interfering with the interaction between *BRD4* and acetyl lysine on target proteins (17). Currently, more than 30 targeted inhibitors have shown significant inhibitory effects against various tumors in preclinical and clinical trials (18). Therefore, BRD2, BRD3, and BRD4 may be biomarkers for some cancers, and the development of their inhibitors may be a potential strategy for cancer treatment.

The role of *BRD2*, *BRD3*, and *BRD4* in ACC is not well understood. Therefore, this study systematically analyzed the expression, gene regulatory network, prognostic value, and target prediction of *BRD2*, *BRD3*, and *BRD4* in ACC patients, elucidated the association between BET family and ACC, and identified potential new targets for ACC therapy.

## 2 Materials and methods

### 2.1 UALCAN analysis

UALCAN (http://ualcan.path.uab.edu/analysis.html) is an online professional database for analyzing tumor gene expression levels. We used UALCAN to analyze the expression levels of *BRD2*, *BRD3*, and *BRD4* in ACC patients with different cancer stages. The “Expression Analysis” module of UALCAN database was used to analyze TCGA gene expression data, and the screening criteria were set as: (1) gene: *BRD2*, *BRD3*, and *BRD4*; (2) dataset: ACC; (3) 77 ACC patients (9 in stage 1, 37 in stage 2, 16 in stage 3, and 15 in stage 4); and threshold setting conditions: *P*-value cutoff = 0.05. The Student’s *t*-test was used for comparative analysis.

### 2.2 GEPIA

Gene Expression Profiling (GEPIA) (http://gepia.cancer-pku.cn/index.html) is a free online platform for analyzing the correlation of gene expression levels with the tumor pathological stage and prognostic value. We used GEPIA to analyze the pathological stage correlation and prognostic value of the expression level of *BRD2*, *BRD3*, and *BRD4* in patients with ACC. The screening criteria were: (1) Gene: *BRD2*, *BRD3*, and *BRD4*; (2) dataset: ACC; and (3) threshold setting conditions: *P*-value cutoff = 0.05. The Student’s *t*-test was used to analyze the expression of *BRD2*, *BRD3*, *BRD4*, and the pathological stage of ACC. The Kaplan–Meier curve was used to analyze the prognosis of patients with ACC.

### 2.3 cBioPortal analysis

cBioPortal (http://cbioportal.org) is an online professional database used to analyze genetic alterations in tumors. We used the cBioPortal database to analyze genetic alterations in *BRD2*, *BRD3*, *BRD4*, and their neighboring genes. A total of 75 ACC samples were analyzed, and mRNA expression z-scores were obtained relative to all samples (log RNA Seq V2 RSEM) using a z-score threshold of ±2.0.

### 2.4 STRING analysis

STRING (https://string-db.org/cgi/input.pl) is an online professional database for analyzing protein-protein interactions (PPI). We used STRING to build a low-confidence level (0.150) PPI network interaction and screening criteria for species defined as humans.

### 2.5 GeneMANIA analysis

GeneMANIA (http://www.genemania.org) is a free professional tool for analyzing gene functions. We used GeneMANIA to explore the function of *BRD2*, *BRD3*, *BRD4*, and the top 50 altered neighboring genes, respectively.

### 2.6 Metascape analysis

Metascape (https://metascape.org) is a professional-free tool for analyzing gene Gene Ontology (GO) functions and Kyoto Encyclopedia of Genes and Genomes (KEGG) pathway enrichment. We used Metascape to analyze the GO function and KEGG pathway enrichment of *BRD2*, *BRD3*, *BRD4*, and their altered neighboring genes in ACC.

### 2.7 TRRUST analysis

TRRUST (https://www.grnpedia.org/trrust/) is an online professional database that analyzes gene transcription regulators. We used the TRRUST database to analyze the transcriptional regulators of *BRD2*, *BRD3*, *BRD4*, and their altered neighboring genes in patients with ACC.

### 2.8 LinkedOmics analysis

LinkedOmics (http://www.linkedomics.org/) is a free online platform for analyzing miRNA target enrichment and differentially-expressed genes (DEGs) associated with tumor genes. We used the LinkedOmics database to analyze the miRNA target enrichment and differentially-expressed genes associated with *BRD2*, *BRD3*, and *BRD4*.

### 2.9 Timer analysis

TIMER (https://cistrome.shinyapps.io/timer/) is a specialized database for systematically analyzing tumor genes associated with infiltrating immune cells. We used TIMER to analyze the correlation between *BRD2*, *BRD3*, and *BRD4* expression with immune cell infiltration levels.

### 2.10 Genomics of drug sensitivity in cancer analysis

The Genomics of Drug Sensitivity in Cancer (http://www.cancerRxgene.org) is a specialized public database for obtaining information on antitumor drug sensitivity. We used this database to find targeted drugs of *BRD2*, *BRD3*, and *BRD4*, and predict their anti-ACC activity.

## 3 Results

### 3.1 BET family expression in patients with ACC

**Figure 1:**
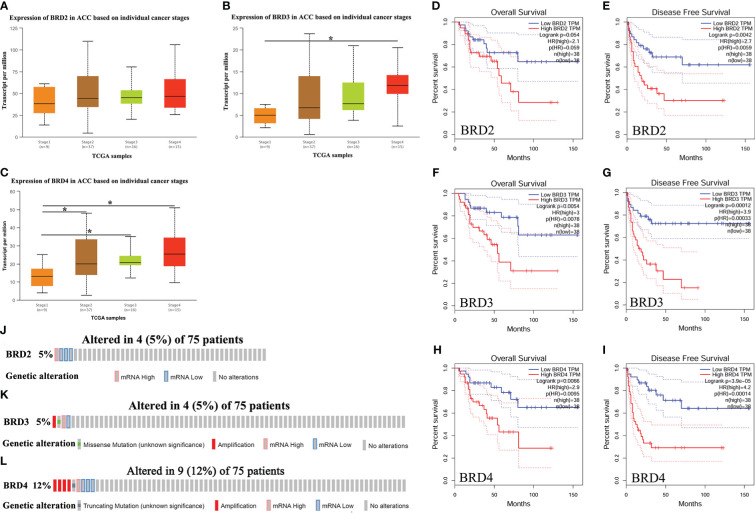
The expression, prognostic value, and genetic alteration of BET family in ACC patients (UALCAN, GEPIA, and cBioPortal). **(A)** The expression of *BRD2* in ACC patients based on individual cancer stages (UALCAN); **(B)** The expression of *BRD3* in ACC patients based on individual cancer stages (UALCAN); **(C)** The expression of *BRD4* in ACC patients based on individual cancer stages (UALCAN); **(D)** The overall survival curve of *BRD2* in patients with ACC (GEPIA); **(E)** The disease-free survival cure of *BRD2* in patients with ACC (GEPIA); **(F)** The overall survival curve of *BRD3* in patients with ACC (GEPIA); **(G)** The disease-free survival cure of *BRD3* in patients with ACC (GEPIA); **(H)** The overall survival curve of *BRD4* in patients with ACC (GEPIA); **(I)** The disease-free survival cure of *BRD4* in patients with ACC (GEPIA). **(J)** Genetic alteration of *BRD2* in ACC (cBioPortal); **(K)** Genetic alteration of *BRD3* in ACC (cBioPortal); **(L)** Genetic alteration of *BRD4* in ACC (cBioPortal). Data are expresssed as mean ± SE. *P < 0.05.

We first compared the expression levels of *BRD2*, *BRD3*, and *BRD4* in ACC patients with different cancer stages and found that the *BRD3* (stage 4) and *BRD4* transcript levels were significantly upregulated in patients with ACC (*P* < 0.05) (**Figures 1B, C**). However, BRD2 transcript levels did not change (**Figures 1A**). In addition, we evaluated the correlation between the differential expression of *BRD2*, *BRD3*, and *BRD4* with the pathological stage of ACC. We found a significant correlation between the expression of *BRD4* and the pathological stage of patients with ACC (*P* = 0.0104) (**Supplementary Figure 1C**). Finally, we used GEPIA to assess the prognostic value of *BRD2*, *BRD3*, and *BRD4* expressions in ACC patients. Our results showed that ACC patients with low *BRD3* and *BRD4* expressions had longer overall survival than those with high *BRD3* (*P* = 0.0054) (**Figure 1F**) and *BRD4* expressions (*P* = 0.0066) (**Figure 1H**). Similarly, ACC patients with low *BRD2*, *BRD3*, and *BRD4* expressions had longer disease-free survival than those with high *BRD2* (*P* = 0.0042) (**Figure 1E**), *BRD3* (*P* = 0.00012) (**Figure 1G**), and *BRD4* expressions (*P* = 3.9e-5) (**Figure 1I**). However, BRD2 expressions had no effect on the overall survival of ACC patients (**Figure 1D**).

### 3.2 Genetic alteration of the BET family in patients with ACC

We further assessed genetic alterations in *BRD2*, *BRD3*, and *BRD4* in 75 patients with ACC using The Cancer Genome Atlas (TCGA). We found that the expression of *BRD2* and *BRD3* was altered by 5% in patients with ACC, with the type of genetic alteration mainly including high and low RNA levels (**Figures 1J, K**). However, the expression of *BRD4* was altered by 12% in patients with ACC, with the type of genetic alteration mainly including truncating mutation, amplification, and high and low RNA levels (**Figure 1L**).

### 3.3 Neighboring gene alteration and BET family interaction network in patients with ACC

We evaluated the alterations in the neighboring genes of *BRD2*, *BRD3*, and *BRD4* in patients with ACC using the cBioPortal. Our results showed a gene alteration frequency of ≥25.00% for 50 of the most frequently altered neighboring genes of *BRD2* and *BRD3* in patients with ACC (**Tables 1**, **2**). However, we found a gene alteration frequency of ≥ 44.44% for the 50 most frequently altered neighboring genes of *BRD4* in patients with ACC (**Table 3**). Furthermore, the most frequently altered neighboring genes of *BRD2*, *BRD3*, and *BRD4* in patients with ACC were *CLDN23* (75.00%), *PLEC* (75.00%), *MAL2* (75.00%), *SYNE1* (75.00%), *GPRIN2* (75.00%), *ADAMTS13* (50.00%), *NOTCH3* (55.56%), *ANGPTL6* (44.44%), and *C19ORF38* (44.44%) (**Tables 1**- **3**).

**Table 1:** The top 50 of *BRD2* neighbor gene alterations in ACC (cBioPortal).

Gene	Altered group	Unaltered group	*p*-Value
*HEATR3*	2 (50.00%)	0 (0.00%)	2.16E-03
*ROBO1*	2 (50.00%)	0 (0.00%)	2.16E-03
*CAPN15*	2 (50.00%)	1 (1.41%)	6.37E-03
*SCN7A*	2 (50.00%)	2 (2.82%)	0.0125
*CLDN23*	3 (75.00%)	10 (14.08%)	0.0152
*PLEC*	3 (75.00%)	12 (16.90%)	0.0236
*MAL2*	3 (75.00%)	13 (18.31%)	0.0287
*ZAR1*	3 (75.00%)	15 (21.13%)	0.0408
*ABCC12*	1 (25.00%)	0 (0.00%)	0.0500
*ABCG1*	1 (25.00%)	0 (0.00%)	0.0500
*ACTG2*	1 (25.00%)	0 (0.00%)	0.0500
*ACTRT1*	1 (25.00%)	0 (0.00%)	0.0500
*ADCY7*	1 (25.00%)	0 (0.00%)	0.0500
*ADGRG1*	1 (25.00%)	0 (0.00%)	0.0500
*ADGRG3*	1 (25.00%)	0 (0.00%)	0.0500
*ADGRG5*	1 (25.00%)	0 (0.00%)	0.0500
*ADH6*	1 (25.00%)	0 (0.00%)	0.0500
*AKTIP*	1 (25.00%)	0 (0.00%)	0.0500
*AMFR*	1 (25.00%)	0 (0.00%)	0.0500
*ANKRD26P1*	1 (25.00%)	0 (0.00%)	0.0500
*ANKRD29*	1 (25.00%)	0 (0.00%)	0.0500
*ANO3*	1 (25.00%)	0 (0.00%)	0.0500
*AP1G1*	1 (25.00%)	0 (0.00%)	0.0500
*AQP4*	1 (25.00%)	0 (0.00%)	0.0500
*ARHGAP23P1*	1 (25.00%)	0 (0.00%)	0.0500
*ARL2BP*	1 (25.00%)	0 (0.00%)	0.0500
*ART5*	1 (25.00%)	0 (0.00%)	0.0500
*ATXN1L*	1 (25.00%)	0 (0.00%)	0.0500
*B3GALT5-AS1*	1 (25.00%)	0 (0.00%)	0.0500
*BACE2*	1 (25.00%)	0 (0.00%)	0.0500
*C16ORF78*	1 (25.00%)	0 (0.00%)	0.0500
*C16ORF87*	1 (25.00%)	0 (0.00%)	0.0500
*C2CD2*	1 (25.00%)	0 (0.00%)	0.0500
*CAPNS2*	1 (25.00%)	0 (0.00%)	0.0500
*CASC16*	1 (25.00%)	0 (0.00%)	0.0500
*CBS*	1 (25.00%)	0 (0.00%)	0.0500
*CCDC113*	1 (25.00%)	0 (0.00%)	0.0500
*CCDC71*	1 (25.00%)	0 (0.00%)	0.0500
*CCL17*	1 (25.00%)	0 (0.00%)	0.0500
*CCL22*	1 (25.00%)	0 (0.00%)	0.0500
*CDPF1*	1 (25.00%)	0 (0.00%)	0.0500
*CEP68*	1 (25.00%)	0 (0.00%)	0.0500
*CES1*	1 (25.00%)	0 (0.00%)	0.0500
*CES1P1*	1 (25.00%)	0 (0.00%)	0.0500
*CFAP20*	1 (25.00%)	0 (0.00%)	0.0500
*CHST4*	1 (25.00%)	0 (0.00%)	0.0500
*CIAPIN1*	1 (25.00%)	0 (0.00%)	0.0500
*CLDN14*	1 (25.00%)	0 (0.00%)	0.0500
*CMTR2*	1 (25.00%)	0 (0.00%)	0.0500
*CNEP1R1*	1 (25.00%)	0 (0.00%)	0.0500

**Table 2:** The top 50 of *BRD3* neighbor gene alterations in ACC (cBioPortal).

Gene	Altered group	Unaltered group	*p*-Value
*ADAMTS13*	2 (50.00%)	0 (0.00%)	2.16E-03
*CCDC157*	2 (50.00%)	0 (0.00%)	2.16E-03
*CRELD2*	2 (50.00%)	0 (0.00%)	2.16E-03
*GOLGA2*	2 (50.00%)	0 (0.00%)	2.16E-03
*GRIN1*	2 (50.00%)	0 (0.00%)	2.16E-03
*KCNT1*	2 (50.00%)	0 (0.00%)	2.16E-03
*ODF3B*	2 (50.00%)	0 (0.00%)	2.16E-03
*PCNX2*	2 (50.00%)	0 (0.00%)	2.16E-03
*STKLD1*	2 (50.00%)	0 (0.00%)	2.16E-03
*SURF6*	2 (50.00%)	0 (0.00%)	2.16E-03
*TMEM203*	2 (50.00%)	0 (0.00%)	2.16E-03
*SYNE1*	3 (75.00%)	5 (7.04%)	3.15E-03
*CDK5RAP2*	2 (50.00%)	1 (1.41%)	6.37E-03
*GAPVD1*	2 (50.00%)	1 (1.41%)	6.37E-03
*NOXA1*	2 (50.00%)	1 (1.41%)	6.37E-03
*PABPC1*	2 (50.00%)	1 (1.41%)	6.37E-03
*PLXNB2*	2 (50.00%)	1 (1.41%)	6.37E-03
*XBP1*	2 (50.00%)	1 (1.41%)	6.37E-03
*CARNMT1*	2 (50.00%)	2 (2.82%)	0.0125
*FBN2*	2 (50.00%)	2 (2.82%)	0.0125
*GOLGA1*	2 (50.00%)	2 (2.82%)	0.0125
*KCNJ11*	2 (50.00%)	2 (2.82%)	0.0125
*NR5A1*	2 (50.00%)	2 (2.82%)	0.0125
*PHACTR2*	2 (50.00%)	2 (2.82%)	0.0125
*SECISBP2*	2 (50.00%)	2 (2.82%)	0.0125
*GPRIN2*	3 (75.00%)	10 (14.08%)	0.0152
*CHEK2*	2 (50.00%)	3 (4.23%)	0.0204
*MTRFR*	2 (50.00%)	3 (4.23%)	0.0204
*SPRR3*	2 (50.00%)	3 (4.23%)	0.0204
*ZC3H4*	2 (50.00%)	3 (4.23%)	0.0204
*CEMIP2*	2 (50.00%)	4 (5.63%)	0.0301
*PRR21*	2 (50.00%)	4 (5.63%)	0.0301
*PWWP2B*	2 (50.00%)	4 (5.63%)	0.0301
*LZTR1*	2 (50.00%)	5 (7.04%)	0.0413
*PLXNB3*	2 (50.00%)	5 (7.04%)	0.0413
*ABO*	1 (25.00%)	0 (0.00%)	0.0500
*ACR*	1 (25.00%)	0 (0.00%)	0.0500
*ADAMTSL2*	1 (25.00%)	0 (0.00%)	0.0500
*ADM2*	1 (25.00%)	0 (0.00%)	0.0500
*ADNP2*	1 (25.00%)	0 (0.00%)	0.0500
*AGPAT2*	1 (25.00%)	0 (0.00%)	0.0500
*AGPAT3*	1 (25.00%)	0 (0.00%)	0.0500
*AHSA1*	1 (25.00%)	0 (0.00%)	0.050
*AIF1L*	1 (25.00%)	0 (0.00%)	0.0500
*AK1*	1 (25.00%)	0 (0.00%)	0.0500
*AK8*	1 (25.00%)	0 (0.00%)	0.0500
*ALG12*	1 (25.00%)	0 (0.00%)	0.0500
*ALPP*	1 (25.00%)	0 (0.00%)	0.0500
*ANAPC2*	1 (25.00%)	0 (0.00%)	0.0500
*ANGPTL2*	1 (25.00%)	0 (0.00%)	0.0500

**Table 3:** The top 50 of *BRD4* neighbor gene alterations in ACC (cBioPortal).

Gene	Altered group	Unaltered group	*p*-Value
*NOTCH3*	5 (55.56%)	1 (1.52%)	4.17E-05
*ANGPTL6*	4 (44.44%)	0 (0.00%)	1.04E-04
*C19ORF38*	4 (44.44%)	0 (0.00%)	1.04E-04
*C3P1*	4 (44.44%)	0 (0.00%)	1.04E-04
*CARM1*	4 (44.44%)	0 (0.00%)	1.04E-04
*CASP14*	4 (44.44%)	0 (0.00%)	1.04E-04
*CHERP*	4 (44.44%)	0 (0.00%)	1.04E-04
*COL5A3*	4 (44.44%)	0 (0.00%)	1.04E-04
*CYP4F22*	4 (44.44%)	0 (0.00%)	1.04E-04
*CYP4F23P*	4 (44.44%)	0 (0.00%)	1.04E-04
*CYP4F24P*	4 (44.44%)	0 (0.00%)	1.04E-04
*CYP4F3*	4 (44.44%)	0 (0.00%)	1.04E-04
*CYP4F8*	4 (44.44%)	0 (0.00%)	1.04E-04
*DNMT1*	4 (44.44%)	0 (0.00%)	1.04E-04
*EPHX3*	4 (44.44%)	0 (0.00%)	1.04E-04
*FDX2*	4 (44.44%)	0 (0.00%)	1.04E-04
*ICAM3*	4 (44.44%)	0 (0.00%)	1.04E-04
*ICAM4*	4 (44.44%)	0 (0.00%)	1.04E-04
*ICAM5*	4 (44.44%)	0 (0.00%)	1.04E-04
*ILVBL*	4 (44.44%)	0 (0.00%)	1.04E-04
*LDLR*	4 (44.44%)	0 (0.00%)	1.04E-04
*LINC00661*	4 (44.44%)	0 (0.00%)	1.04E-04
*LINC00905*	4 (44.44%)	0 (0.00%)	1.04E-04
*MRPL4*	4 (44.44%)	0 (0.00%)	1.04E-04
*NACC1*	4 (44.44%)	0 (0.00%)	1.04E-04
*OLFM2*	4 (44.44%)	0 (0.00%)	1.04E-04
*OR10H2*	4 (44.44%)	0 (0.00%)	1.04E-04
*OR10H4*	4 (44.44%)	0 (0.00%)	1.04E-04
*OR10H5*	4 (44.44%)	0 (0.00%)	1.04E-04
*OR1I1*	4 (44.44%)	0 (0.00%)	1.04E-04
*OR7C1*	4 (44.44%)	0 (0.00%)	1.04E-04
*OR7C2*	4 (44.44%)	0 (0.00%)	1.04E-04
*PDE4A*	4 (44.44%)	0 (0.00%)	1.04E-04
*PGLYRP2*	4 (44.44%)	0 (0.00%)	1.04E-04
*PPAN*	4 (44.44%)	0 (0.00%)	1.04E-04
*RASAL3*	4 (44.44%)	0 (0.00%)	1.04E-04
*RN7SL192P*	4 (44.44%)	0 (0.00%)	1.04E-04
*SHFL*	4 (44.44%)	0 (0.00%)	1.04E-04
*SLC1A6*	4 (44.44%)	0 (0.00%)	1.04E-04
*SNORD105*	4 (44.44%)	0 (0.00%)	1.04E-04
*SNORD105B*	4 (44.44%)	0 (0.00%)	1.04E-04
*TIMM29*	4 (44.44%)	0 (0.00%)	1.04E-04
*TPM4*	4 (44.44%)	0 (0.00%)	1.04E-04
*TYK2*	4 (44.44%)	0 (0.00%)	1.04E-04
*UCA1*	4 (44.44%)	0 (0.00%)	1.04E-04
*YIPF2*	4 (44.44%)	0 (0.00%)	1.04E-04
*ZGLP1*	4 (44.44%)	0 (0.00%)	1.04E-04
*AKAP8L*	4 (44.44%)	1 (1.52%)	4.89E-04
*CDC37*	4 (44.44%)	1 (1.52%)	4.89E-04
*CYP4F11*	4 (44.44%)	1 (1.52%)	4.89E-04

**Figure 2:**
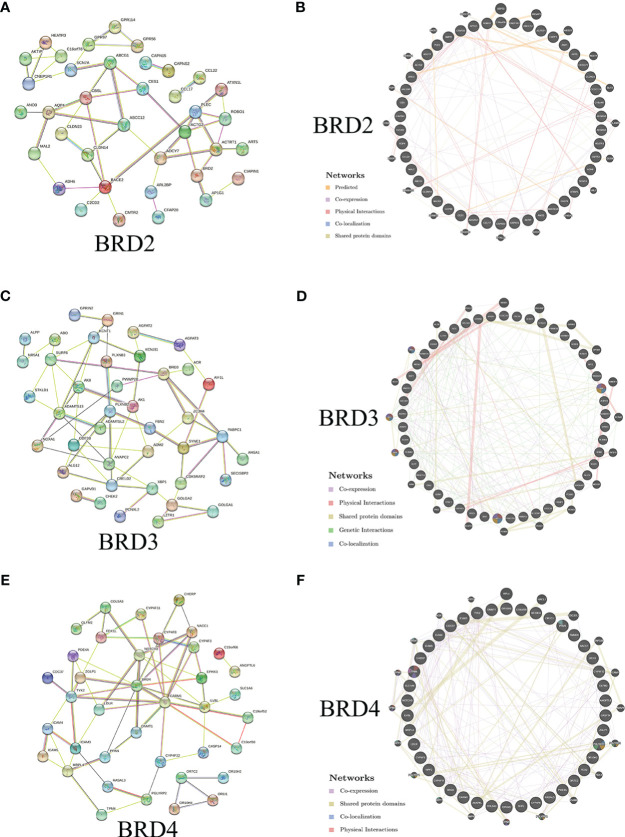
Interaction analyses of BET family and their neighboring genes in ACC (STRING and GeneMANIA). **(A)** PPI network of *BRD2* and its neighboring genes in patients with ACC (STRING); **(B)** Network analyses of *BRD2* and its neighboring genes in patients with ACC (GeneMANIA); **(C)** PPI network of *BRD3* and its neighboring genes in patients with ACC (STRING); **(D)** Network analyses of *BRD3* and its neighboring genes in patients with ACC (GeneMANIA); **(E)** PPI network of *BRD4* and its neighboring genes in patients with ACC (STRING); **(F)** Network analyses of *BRD4* and its neighboring genes in patients with ACC (GeneMANIA).

We further evaluated the potential interactions between *BRD2*, *BRD3*, *BRD4*, and their neighboring genes. We found that 37 nodes and 100 edges were obtained in the constructed PPI network of *BRD2* and its neighboring genes in patients with ACC (**Figure 2A**). Furthermore, *BRD2* and its neighboring genes were linked to a complex interaction network (66 genes and 118 edges) through prediction, co-expression, physical interactions, co-localization, and shared protein domains (**Figure 2B**). We found that 41 nodes and 126 edges were obtained in the constructed PPI network of *BRD3* and its neighboring genes in patients with ACC (**Figure 2C**). *BRD3* and its neighboring genes were linked to a complex interaction network (69 genes and 221 edges) through co-expression, physical interactions, shared protein domains, genetic interactions, and co-localization (**Figure 2D**). Our results showed that 38 nodes and 128 edges were obtained in the constructed PPI network of *BRD4* and its neighboring genes in patients with ACC (**Figure 2E**). *BRD4* and its neighboring genes were linked to a complex interaction network (62 genes and 233 edges) through co-expression, physical interactions, shared protein domains, and colocalization (**Figure 2F**).

### 3.4 GO and KEGG pathway enrichment analyses

**Figure 3:**
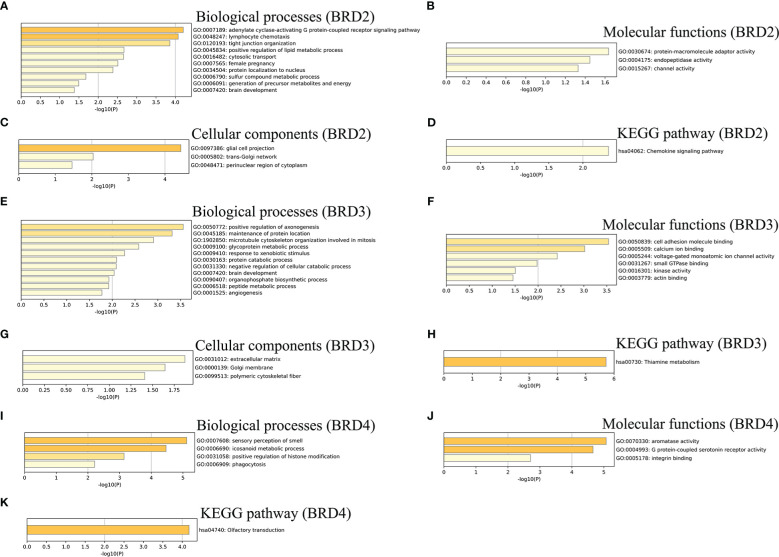
GO function and KEGG pathways enrichment analyses of BET family and their neighboring genes in ACC (metascape). **(A)** Biological processes of *BRD2* and its neighboring genes; **(B)** Molecular functionsf *BRD2* and its neighboring genes; **(C)** Cellular components of *BRD2* and its neighboring genes; **(D)** KEGG pathway analysis of *BRD2* and its neighboring genes; **(E)** Biological processes of *BRD3* and its neighboring genes; **(F)** Molecular functionsf *BRD3* and its neighboring genes; **(G)** Cellular components of *BRD3* and its neighboring genes; **(H)** KEGG pathway analysis of *BRD3* and its neighboring genes; **(I)** Biological processes of *BRD4* and its neighboring genes; **(J)** Molecular functionsf *BRD4* and its neighboring genes; **(K)** KEGG pathway analysis of *BRD4* and its neighboring genes.

We further performed the GO and KEGG pathway enrichment analyses of *BRD2*, *BRD3*, *BRD4*, and the top 50 altered neighboring genes in ACC patients using Metascape. We found that biological processes related to *BRD2* and its neighboring genes in patients with ACC mainly include adenylate cyclase-activating G protein-coupled receptor signaling pathway, generation of precursor metabolites and energy, and lymphocyte chemotaxis (**Figure 3A**). Their molecular functions include protein-macromolecule adaptor activity, endopeptidase activity, and channel activity (**Figure 3B**). Their cellular components related to *BRD2* and its neighboring genes in patients with ACC mainly include glial cell projection, trans-Golgi network, and perinuclear region of cytoplasm (**Figure 3C**). Chemokine signaling pathway was found to be enriched as per the KEGG pathway analysis (**Figure 3D**). Furthermore, our results showed that biological processes related to *BRD3* and its neighboring genes in patients with ACC mainly include angiogenesis, peptide metabolic process, and microtubule cytoskeleton organization involved in mitosis (**Figure 3E**). Their molecular functions include cell adhesion molecule binding, calcium ion binding, and voltage-gated monoatomic ion channel activity (**Figure 3F**). Their cellular components related to *BRD3* and its neighboring genes in patients with ACC mainly include extracellular matrix, Golgi membrane, and polymeric cytoskeletal fiber (**Figure 3G**). Thiamine metabolism was found to be enriched as per the KEGG pathway analysis (**Figure 3H**). Finally, we found that biological processes related to *BRD4* and its neighboring genes in patients with ACC mainly include sensory perception of smell, icosanoid metabolic processes, positive regulation of histone modification, and phagocytosis (**Figure 3I**). Their molecular functions include aromatase activity, G protein-coupled serotonin receptor activity, and integrin binding (**Figure 3J**). Olfactory transduction was found to be enriched as per the KEGG pathway analysis (**Figure 3K**).

### 3.5 Transcription factor and miRNA targets regulating BET family expression in patients with ACC

We used TRRUST to analyze the key regulatory factors of *BRD2*, *BRD3*, and *BRD4* in patients with ACC. Our results showed that SP1 may be the key transcription factor of *BRD2* and its neighboring genes in patients with ACC (*P* < 0.05) (**Table 4**). Among them, *ADH6*, *BACE2*, *CBS*, *CES1*, and *CHST4* may be the main regulatory genes of SP1. Furthermore, we found that NPM1, STAT3, and TP53 may be the key transcription factors of *BRD4* and its neighboring genes in patients with ACC (*P* < 0.05) (**Table 4**). Among them, *BRD4* and *DNMT1* may be the main regulatory genes of NPM1. STAT3 regulates the functions of *DNMT1* and *TYK2*. Moreover, *CARM1* and *DNMT1* may be the main regulatory genes for TP53. Next, we analyzed the miRNA targets of *BRD2*, *BRD3*, and *BRD4* using LinkedOmics. The top three miRNA targets of *BRD2* in patients with ACC may be (ACACTAC) miR-142-3P, (GTGACTT) miR-224, and (TAATGTG) miR-323 (FDP < 0.01) (**Table 5**). (GAGCCTG) miR-484, (GCACCTT) miR-18A, miR-18B, and (GTCTTCC) miR-7 may be the top three miRNA targets of *BRD3* in patients with ACC (FDP < 0.05) (**Table 5**). (TGCACTT) miR-519C, miR-519B, miR-519A, (GCACTTT) miR-17-5P, miR-20A, miR-106A, miR-106B, miR-20B, miR-519D, and (ATGTACA) miR-493 may be the top three miRNA targets of *BRD4* in patients with ACC (FDP = 0) (**Table 5**).

**Table 4:** Key regulated factor of BET family and the top 50 neighbor altered gene in ACC (TRRUST).

Gene	Key TF	Description	Regulated gene	*P*-value
*BRD2*	SP1	Sp1 transcription factor	*ADH6*, *BACE2*, *CBS*, *CES1*, *CHST4*	0.00466
*BRD4*	NPM1	nucleophosmin (nucleolar phosphoprotein B23, numatrin)	*BRD4*, *DNMT1*	0.000226
	STAT3	signal transducer and activator of transcription 3 (acute-phase response factor)	*DNMT1*, *TYK2*	0.0348
	TP53	tumor protein p53	*CARM1*, *DNMT1*	0.0451

**Table 5:** The top three miRNA target of BET family in ACC (LinkedOmics).

Gene	Gene Set	Leandig Edge Number	P-value	FDR
BRD2	ACACTAC,miR-142-3P	50	0	0
	GTGACTT,miR-224	37	0	0
	TAATGTG,miR-323	53	0	0.001066
BRD3	GAGCCTG,miR-484	27	0	0.00239
	GCACCTT,miR-18A,miR-18B	22	0	0.008801
	GTCTTCC,miR-7	39	0	0.010561
BRD4	TGCACTT,miR-519C,miR-519B,miR-519A	151	0	0
	GCACTTT,miR-17-5P,miR-20A,miR-106A,miR-106B,miR-20B,miR-519D	199	0	0
	ATGTACA,miR-493	92	0	0

### 3.6 Correlation of differentially expressed genes and BET family expression in patients with ACC

**Figure 4:**
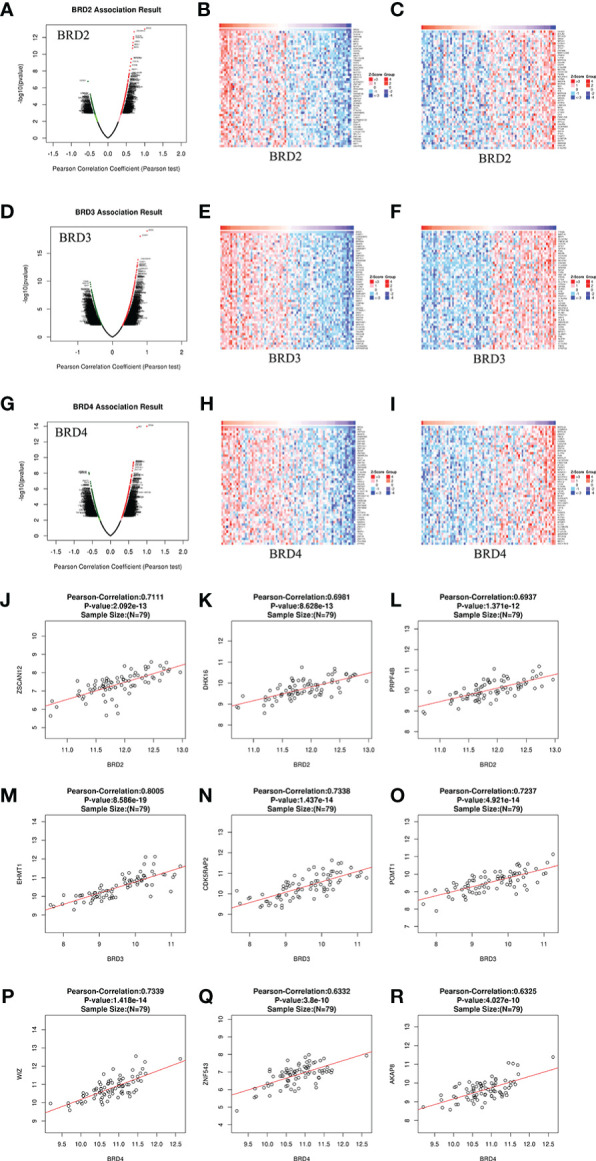
Genes differentially expressed in correlation with BET family expression in ACC (LinkedOmics). **(A)** Pearson test was used to analyze correlations between *BRD2* expression and genes differentially expressed in patients with ACC; **(B, C)** Heatmaps showing genes positively and negatively correlated, respectively, with *BRD2* in patients with ACC (top 50 genes); **(D)** Pearson test was used to analyze correlations between *BRD3* expression and genes differentially expressed in patients with ACC; **(E, F)** Heatmaps showing genes positively and negatively correlated, respectively, with *BRD3* in patients with ACC (top 50 genes); **(G)** Pearson test was used to analyze correlations between *BRD4* expression and genes differentially expressed in patients with ACC; **(H, I)** Heatmaps showing genes positively and negatively correlated, respectively, with *BRD4* in patients with ACC (top 50 genes). **(J, K, L)** The scatter plots show Pearson’s correlation of *BRD2* expression with expression of *ZSCAN12*, *DHX16*, and *PRPF4B*, respectively, in patients with ACC; **(M, N, O)** The scatter plots show Pearson’s correlation of *BRD3* expression with expression of *EHMT1*, *CDK5RAP2*, and *POMT1*, respectively, in patients with ACC; **(P, Q, R)** The scatter plots show Pearson’s correlation of *BRD4* expression with expression of *WIZ*, *ZNF543*, and *AKAP8*, respectively, in patients with ACC.

We analyzed the mRNA sequencing data from 79 patients with ACC using the LinkedOmics TCGA database. In patients with ACC, 19,339 genes were closely related to *BRD2*, *BRD3*, and *BRD4* (**Figures 4A, D, and G**). Among them, positive and negative genes (9,647 and 9,692, 9,115 and 10,224, 10,028 and 9,311) were found correlate with *BRD2*, *BRD3*, and *BRD4* expressions, respectively (**Figures 4A, 4D and G**). Moreover, we found that 50 genes had a notable positive or negative correlation with *BRD2*, *BRD3*, and *BRD4* expressions in patients with ACC (*P* < 0.05) (**Figures 4B, C, E, F, H, and I**). Among them, *BRD2* expression was strongly positively associated with the expression of *ZSCAN12* (Pearson correlation coefficient (PCO) = 0.7111, *P* = 2.092e–13; **Figure 4J**), *DHX16* (PCO = 0.6981, *P* = 8.628e–13; **Figure 4K**), and *PRPF4B* (PCO = 0.6937, *P* = 1.371e–12; **Figure 4L**). However, the expression of *BRD3* was positively associated with the expression of *EHMT1* (PCO = 0.8005, *P* = 8.586e–19; **Figure 4M**), *CDK5RAP2* (Pearson correlation = 0.7338, *P* = 1.437e–14; **Figure 4N**), and *POMT1* (PCO = 0.7237, *P* = 4.921e–14; **Figure 4O**). Furthermore, *WIZ* (PCO = 0.7339, *P* = 1.418e-14; **Figure 4P**), *ZNF543* (PCO = 0.6332, *P* = 3.8e-10; **Figure 4Q**), and *AKAP8* (PCO = 0.6325, *P* = 4.027e-10; **Figure 4R**) were the top three genes whose expressions were positively correlated with the expression of *BRD4*.

### 3.7 Immune cell infiltration and BET family expression in patients with ACC

**Figure 5:**
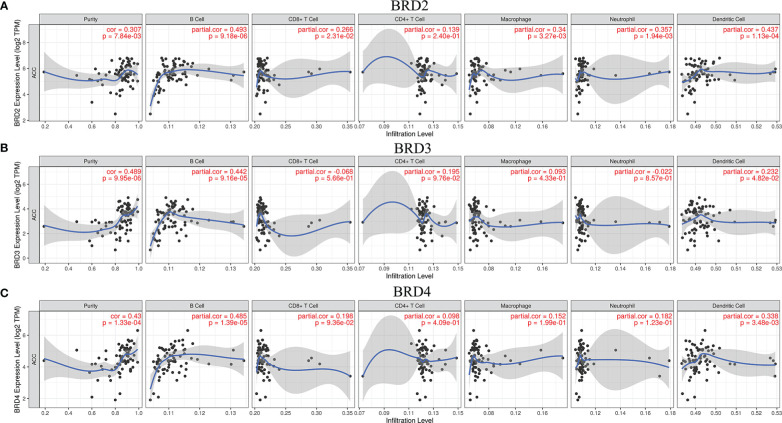
The correlation between BET family expression and immune cell infiltration levels in ACC (TIMER). **(A)** *BRD2*; **(B)** *BRD3*; **(C)** *BRD4*.

We used TIMER to evaluate the relationship between immune cell infiltration levels and *BRD2*, *BRD3*, and *BRD4* expressions in patients with ACC. Our results showed that the expression levels of *BRD2*, *BRD3*, and *BRD4* in patients with ACC were positively correlated mainly with B-cell and dendritic cell infiltration levels (*P*<0.05; **Figures 5A-C**).

### 3.8 BET family targeting Drugs

**Figure 6:**
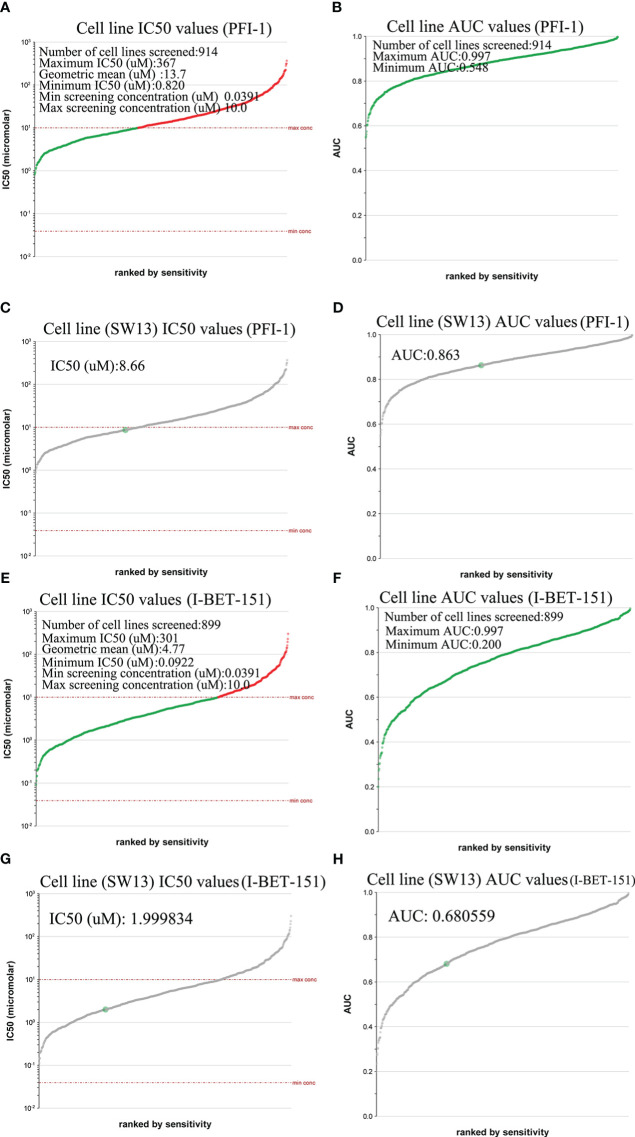
IC50 evaluation of PFI-1 and I-BET-151 in different tissue types of cancer (Genomics of Drug Sensitivity in Cancer). **(A)** Cell line IC50 values of PFI-1; **(B)** Cell line AUC values of PFI-1; **(C)** SW13 cell line IC50 values of PFI-1; **(D)** SW13cell line AUC values of PFI-1. **(E)** Cell line IC50 values of I-BET-151; **(F)** Cell line AUC values of I-BET-151; **(G)** SW13 Cell line IC50 values of I-BET-151; **(H)** SW13cell line AUC values of I-BET-151.

We used the Genomics of Drug Sensitivity in Cancer database to evaluate the inhibitory effect of *BRD4*-targeted drug PFI-1 and (*BRD2*, *BRD3*, and *BRD4*)-targeted drug I-BET-151 on ACC cell lines. PFI-1 inhibited 914 cell lines with area under the curve (AUC) values greater than 0.548 (**Figure 6B**). It had a good inhibitory effect on these cell lines (0.820 ≤ IC50 (μM) ≤ 367) (**Figure 6A**). Furthermore, we found that PFI-1 had good inhibitory effect on SW13 (a cell line of ACC) (AUC values =0.863, IC50 (μM) = 8.66) (**Figures 6C, D**). However, I-BET-151 inhibited 899 cell lines with area under the curve (AUC) values greater than 0.200 (**Figure 6F**). It had a good inhibitory effect on these cell lines (0.0922 ≤ IC50 (μM) ≤ 301) (**Figure 6E**). Furthermore, we found that I-BET-151 had a good inhibitory effect on SW13 (AUC values =0.680559, IC50 (μM) = 1.999834) (**Figures 6G, H**).

## 4 Discussion

The expressions of *BRD2* and *BRD3* in patients with ACC still remain unclear. *BRD4* expression is reported to be significantly upregulated in ACC (19). However, the expression of *BRD4* has not been reported for the individual cancer stages of patients with ACC. We first compared the expression levels of *BRD2*, *BRD3*, and *BRD4* in ACC patients with different cancer stages and found that *BRD3* and *BRD4* transcript levels were significantly upregulated in patients with ACC. Furthermore, we found a significant positive correlation between the expression of *BRD4* and the pathological stage of patients with ACC. In cancer patients with high *BRD4* expression, increased BRD4 activity is associated with higher expression of oncogenes such as *MYC*, *NOTCH3*, and *NRG1* (20). *BRD4*-driven oncogenes promote tumor cell proliferation, metastasis, and increased chemoresistance (20). Our results also revealed that the expression of *BRD2*, *BRD3*, and *BRD4* was altered by 5%, 5%, and 12%, respectively, in patients with ACC, with the type of genetic alteration mainly including high and low RNA levels. Increased expression of *BRD3* and *BRD4* caused by genetic changes may also be an important factor. However, this requires further investigation. Finally, we assessed the prognostic value of *BRD2*, *BRD3*, and *BRD4* expression in ACC patients. Our results showed that ACC patients with low *BRD2*, *BRD3*, and *BRD4* expression had longer survival than those with high *BRD2*, *BRD3*, and *BRD4* expression. *BRD2*, *BRD3*, and *BRD4* may serve as potential prognostic markers in patients with ACC.

Our results showed a gene alteration frequency of ≥25.00%, ≥25.00%, and ≥44.44% for the 50 most frequently altered neighboring genes of *BRD2*, *BRD3*, and *BRD4*, respectively, in patients with ACC. We further evaluated the potential interactions between *BRD2*, *BRD3*, *BRD4*, and their neighboring genes. We found that *BRD2*, *BRD3*, *BRD4*, and their neighboring genes were linked to a complex interaction network through co-expression, physical interactions, and shared protein domains. Next, we evaluated the functions of *BRD2*, *BRD3*, and *BRD4* and their neighboring genes. Our results showed that the biological processes related to *BRD2* and its neighboring genes mainly include adenylate cyclase-activating G protein-coupled receptor signaling pathway, generation of precursor metabolites and energy, and lymphocyte chemotaxis. These biological processes affecting tumor proliferation, invasion, and metastasis have been reported (21–23). Furthermore, the biological processes related to *BRD3* and its neighboring genes mainly include angiogenesis and microtubule cytoskeleton organization involved in mitosis. Abnormal angiogenesis and mitosis are the key factors of tumor occurrence and development (24, 25). Moreover, our results showed that the biological processes related to *BRD4* and its neighboring genes mainly include positive regulation of histone modification and phagocytosis. Histone modification is a reversible process mediated by epigenetic enzymes (26). Histone methylation and acetylation are two important chemical modifications. They play important roles in transcriptional activation/inactivation, chromosomal packaging, and DNA damage/repair (27). The molecular functions of *BRD2* and its neighboring genes include endopeptidase activity and channel activity. Various endopeptidase activities affect tumorigenesis and prognosis. Prolyl endopeptidase is a serine peptidase involved in the differentiation, development, and proliferation of various tissues. Recent studies have shown that the expression and activity of this cytoplasmic enzyme increases in colorectal cancer and affects the prognosis of colon cancer patients (28). The molecular functions of *BRD3* and its neighboring genes include cell adhesion molecule binding, calcium ion binding, and voltage-gated monoatomic ion channel activity. Cell adhesion is a key mediator of cancer progression and promotes cancer hallmarks including immune escape and metastatic spread (29). Calcium (Ca) ion is a key secondary messenger in excitable and unexcitable cells. Ca signaling has received limited attention as a potential target for anticancer therapy (30). However, the molecular functions of *BRD4* and its neighboring genes include aromatase activity, G protein-coupled serotonin receptor activity, and integrin binding. Recently, it has been proved that ACC cell overexpress aromatase and estrogen receptor, and estrogen synthesized by aromatase can enhance the proliferation of ACC cells (31). ACC can cause Cushing’s syndrome. However, some studies have shown that several receptors, including G protein-coupled serotonin receptors, are frequently co-expressed in patients with Cushing’s syndrome (32). Integrins are cell-adhesion receptors. However, integrin expression patterns are frequently altered in cancer. The expression of certain integrins is associated with increased metastasis and decreased cancer (33). Chemokine signaling pathway, thiamine metabolism, and olfactory transduction were enriched as per the KEGG pathway analysis for *BRD2*, *BRD3*, *BRD4*, and their neighboring genes. The tumor microenvironment consists of stromal cells and tumor cells, which interact with each other through complex crosstalk mediated by a variety of growth factors, cytokines, and chemokines. Chemokines play a key role in promoting tumor cell proliferation (34). Thiamine supplementation may contribute to tumor cell survival, proliferation, and chemotherapy resistance. However, some studies suggest that thiamine may exhibit some antitumor effects. The role of thiamine in cancer is controversial (35). Olfactory receptors specifically expressed in certain cancer cells, such as prostate-specific G protein-coupled receptor 1 (PSGR1), are significantly elevated in prostate cancer. NDRG1 affects oncogenic signaling pathways and tumor progression (36). There is no relevant research on whether olfactory receptors are specifically expressed in ACC patients. Taken together, the functions and signaling pathways involving *BRD2*, *BRD3*, *BRD4*, and their neighboring genes may be involved in the occurrence and progression of ACC. The regulation of these genes and signaling pathways may be a potential treatment strategy for ACC.

Next, we explored the transcription factor and miRNA targets of *BRD2*, *BRD3*, and *BRD4* in patients with ACC. We found that SP1, NPM1, STAT3, and TP53 are the key transcription factors of *BRD2*, *BRD4*, and their neighboring genes in patients with ACC. SP1 is a well-known member of the transcription factor family, which plays an important role in cell growth, differentiation, apoptosis, and carcinogenesis (37). However, its role in the ACC has not been reported. Studies have shown that STAT3 can promote angiogenesis in patients with ACC, thereby making STAT3 a selective target for molecular-targeted therapy of ACC (38). ACC is a rare tumor type associated with TP53 mutations. Studies have shown that genetic susceptibility caused by mutations in TP53 is associated with approximately 50% of childhood ACC cases but only 3–6% of adult cases (39). The relationship between NPM1 and ACC has not yet been reported, but may be a new potential therapeutic target for ACC. Furthermore, our results showed that miR-142-3P, miR-224, miR-323, miR-484, miR-18A, miR-18B, miR-7, miR-519C, miR-519B, miR-519A, miR-17-5P, miR-20A, miR-106A, miR-106B, miR-20B, miR-519D, and miR-493 are targets of *BRD2*, *BRD3*, and *BRD4* in patients with ACC. They are associated with tumor cell proliferation, migration, invasion, and drug resistance, and may be promising targets for cancer therapy (40–42). However, their relationship with ACC has not yet been reported. Our results suggest that these transcription factor and miRNA targets of *BRD2*, *BRD3*, *BRD4*, and their neighboring genes may be potentially therapeutic in treating ACC.

We explored the correlation between the differentially expressed genes and BET family expression in patients with ACC. We found that the expression of 19,339 genes was correlated with *BRD2*, *BRD3*, and *BRD4* expression. Among them, *ZSCAN12*, *DHX16*, *PRPF4B*, *EHMT1*, *CDK5RAP2*, *POMT1*, *WIZ*, *ZNF543*, and *AKAP8* were the top nine genes whose expressions were positively correlated with the expression of *BRD2*, *BRD3*, and *BRD4.* Therefore, targeting these cells may provide additional therapy for ACC. Tumor immune infiltration is closely related to the clinical prognosis (43). As expected, the expression levels of *BRD2*, *BRD3*, and *BRD4* in patients with ACC positively correlated with B cell and dendritic cell infiltration levels. Targeting *BRD2*, *BRD3*, and *BRD4* or their related regulatory targets may be a feasible strategy for reducing immune cell infiltration levels in patients with ACC. We further evaluated the inhibitory effect of *BRD4*-targeting drug PFI-1 and (*BRD2*, *BRD3*, and *BRD4*)-targeted drug I-BET-151 on ACC lines. We found that PFI-1 and I-BET-151 inhibited 914 and 899 cancer cell lines, respectively. Among them, I-BET-151 had a better inhibitory effect on the SW13 cell line than PFI-1. In short, PFI-1 and I-BET-151 exhibited broad-spectrum inhibitory effects on cancer cells. Therefore, finding inhibitors of *BRD2*, *BRD3*, and *BRD4* or their regulatory targets may be an important strategy for the treatment of ACC.

In conclusion, this study systematically analyzed the expression, gene regulatory network, prognostic value, and target prediction of *BRD2*, *BRD3*, and *BRD4* in patients with ACC, elucidated the association between *BRD2*, *BRD3*, *BRD4* and ACC, and provided insights into the mechanism and treatment of ACC.

## Data availability statement

The original contributions presented in the study are included in the article/**Supplementary Material**. Further inquiries can be directed to the corresponding authors.

## Author contributions

LD and YS performed data analysis work and aided in writing the manuscript. YS designed the study and assisted in writing the manuscript. QL, ZZ, JC, ZS, QX, XL, YC, LL, JZ edited the manuscript. All authors contributed to the article and approved the submitted version.

## Supplementary material

Supplementary Figure 1 Correlation between the expression of BET family and the pathological stage of ACC (GEPIA). **(A)** *BRD2*; **(B)** *BRD3*; **(C)** *BRD4*.
