# Supplementary figures and images for: Systematic analysis of the BET family in adrenocortical carcinoma: The expression, prognosis, gene regulation network, and regulation targets
Source: Front Endocrinol (Lausanne). 2023 Jan 30;14:1089531. doi: 10.3389/fendo.2023.1089531 (PMC9922706; doi:10.3389/fendo.2023.1089531)

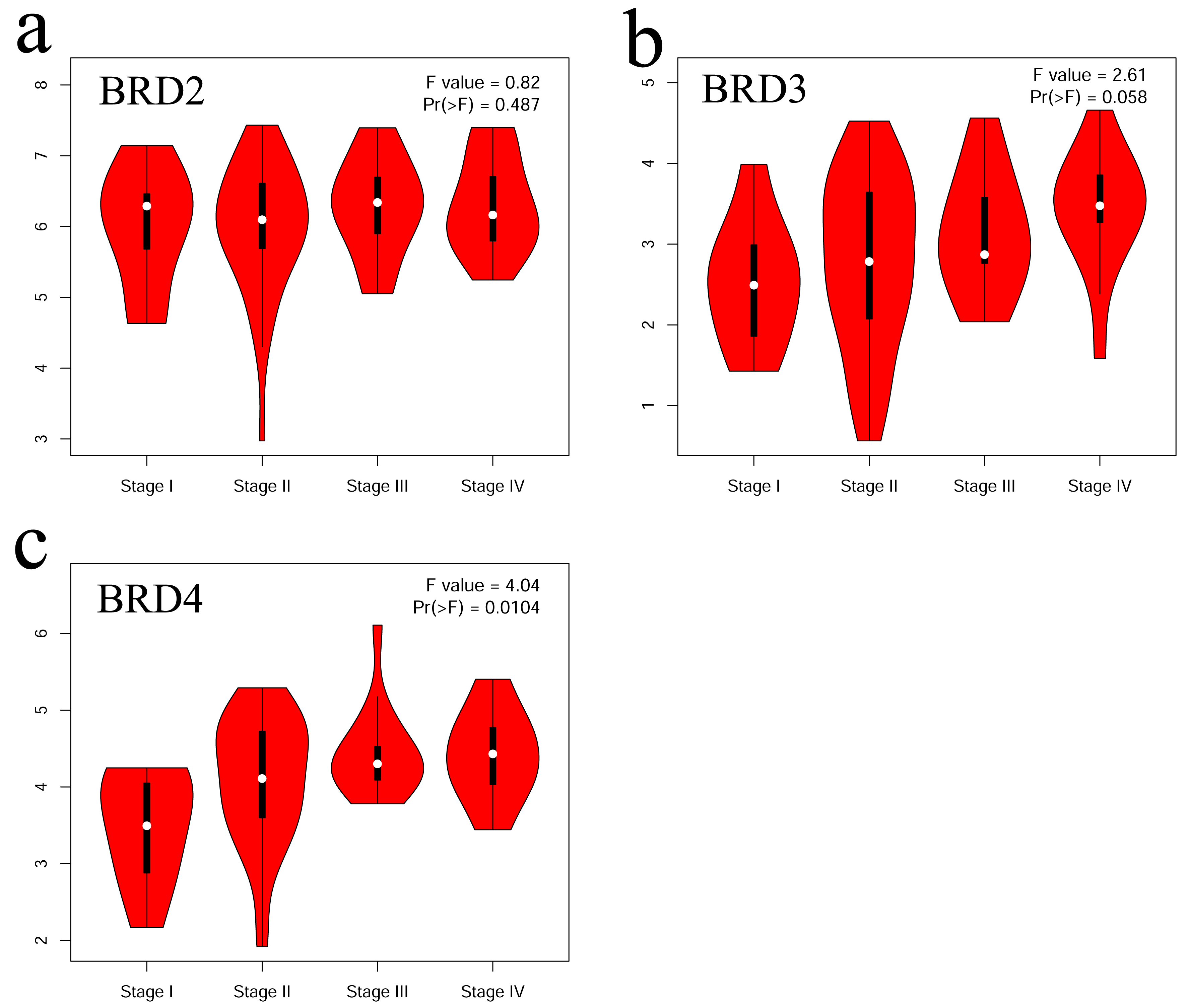

Supplement: Supplementary Figure 1 — Correlation between the expression of BET family and the pathological stage of ACC (GEPIA). (A) BRD2; (B) BRD3; (C) BRD4. [file Image_1.tif]
